# A Multi-Step Computational Workflow for Screening and Prioritizing SHP2-Binding Molecules

**DOI:** 10.3390/ph19050706

**Published:** 2026-04-30

**Authors:** Marina Bilotta, Roberta Rocca, Stefano Alcaro

**Affiliations:** 1 Dipartimento di Scienze Della Salute, Università “Magna Græcia” di Catanzaro, Campus “S. Venuta”, 88100 Catanzaro, Italy; marina.bilotta@studenti.unicz.it (M.B.); alcaro@unicz.it (S.A.); 2 Associazione CRISEA—Centro di Ricerca e Servizi Avanzati per l’Innovazione Rurale, Località Condoleo, 88055 Belcastro, Italy

**Keywords:** SHP2, molecular dynamics, MM/GBSA, virtual screening, structural waters, chemical space modeling, transformer embeddings, ligand prioritization

## Abstract

**Background/Objectives**: SHP2 (*PTPN11*) is a key regulator of RAS/MAPK signaling and a well-validated target in cancer and developmental disorders. Designing ligands for its catalytic site is challenging due to the pocket’s intrinsic flexibility and the presence of conserved structural water molecules critical for ligand recognition, which limits traditional discovery approaches. This study aimed to systematically identify and prioritize novel SHP2-binding candidates using a computational strategy that accounts for these challenges. **Methods**: An integrative computational workflow was applied, combining water-aware docking, large-scale virtual screening of 714,409 compounds, MM/GBSA binding free-energy analysis, AI-driven chemical space modeling using ChemBERTa, and microsecond-scale molecular dynamics (MD) simulations. The high-resolution catalytic PTP domain of SHP2 structure was analyzed to identify conserved water molecules (W711, W716, W726, W776) essential for reproducing the crystallographic binding mode of the reference ligand 3LU. Candidates were prioritized based on docking scores, physicochemical criteria, structural inspection, MM/GBSA energetic profiles, and occupancy of distinct chemical space regions. **Results**: Seven compounds were selected. SwissADME analysis confirmed favorable drug-likeness and GI absorption, with no BBB permeation. ChemBERTa embeddings revealed substantial structural novelty relative to known SHP2 inhibitors. 1 μs molecular dynamics simulations suggested stable binding of compound **4** (2-(3-methyl-2,6-dioxopurin-7-yl)acetate) and persistent interactions with the conserved water network. MM/GBSA evaluation subsequently highlighted its energetically coherent profile. **Conclusions**: The workflow prioritizes compound **4** as a promising and structurally innovative SHP2-binding candidate. This integrative strategy provides a generalizable approach for targeting proteins with flexible pockets, critical water networks, and limited scaffold diversity, offering a roadmap for challenging computational ligand-prioritization projects.

## 1. Introduction

**Figure 1:**
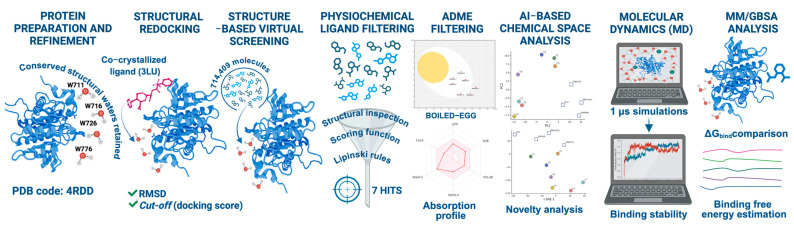
Overview of the integrated computational workflow employed for the identification and prioritization of SHP2-binding ligands.

Protein tyrosine phosphatases (PTPs) [1] are a conserved family of enzymes that regulate oncogenic signaling by removing phosphate groups from phosphotyrosine residues, thereby controlling pathway activation and cellular responses whose dysregulation contributes to cancer development and progression. Historically, PTPs have been considered challenging drug targets due to their shallow, highly conserved, and often solvent-exposed catalytic pockets, which limit the ability of small molecules to achieve both high affinity and selectivity [2]. These structural features increase the likelihood of off-target effects and reduce the efficacy of conventional inhibitors, complicating the development of potent and specific therapeutic agents. Consequently, successful targeting of PTPs requires innovative strategies that explicitly account for pocket flexibility, structural water networks, and dynamic binding environments. Despite these challenges, SHP2 (encoded by *PTPN11*) has emerged as a particularly druggable PTP and a central signaling hub, whose inhibition offers promising therapeutic potential across a broad range of oncogenic pathways. SHP2 modulates the RAS/MAPK cascade by promoting the assembly of signaling complexes and sustaining growth factor-driven cellular proliferation [3]. Gain-of-function mutations in *PTPN11* underlie developmental disorders such as Noonan syndrome, while aberrant SHP2 hyperactivation contributes to tumorigenesis and resistance to targeted therapies [4]. As a result, SHP2 has become a compelling target for anticancer intervention [5]. From a structural and computational perspective, SHP2 poses several non-trivial challenges for ligand discovery. The enzyme comprises N-SH2, C-SH2, and PTP domains that undergo dynamic regulatory motions, including autoinhibition mediated by interdomain interactions [6,7,8]. Accurate modeling of the catalytic site further requires explicit consideration of a set of highly conserved structural water molecules, which stabilize key residues and mediate protein–ligand recognition. These water molecules are integral components of the binding environment; their omission can distort pocket geometry, alter electrostatic potentials, and introduce significant artifacts into docking predictions [9]. Consequently, classical structure-based virtual screening (SBVS) approaches often display limited predictive power for SHP2, where induced-fit effects, water-mediated interactions, and conformational plasticity play decisive roles [10]. Robust computational workflows therefore require the explicit retention of conserved waters [11], post-docking analysis using long-timescale molecular dynamics (MD) simulations [12], energetic evaluation through MM/GBSA calculations [13], and systematic assessment of chemical diversity to avoid rediscovery of known chemotypes [14]. In parallel, recent advances in molecular artificial intelligence, particularly transformer-based language models [15], have transformed the exploration of chemical space. Models such as ChemBERTa encode molecular structures into high-dimensional embedding vectors that capture subtle functional and topological relationships [16], enabling quantitative assessment of scaffold novelty and identification of ligands residing in previously unexplored regions of chemical space [17]. Here, we present a multi-step computational strategy in which docking-derived candidates were first evaluated through MM/GBSA energetic analysis, then assessed by AI-driven chemical space exploration and novelty analysis, and finally validated by explicit-solvent molecular dynamics simulations. This integrated framework enables the systematic prioritization of SHP2-binding candidates and provides a generalizable methodological approach for ligand discovery targeting complex systems characterized by conserved water networks and pronounced conformational plasticity [5,18,19] (Figure 1).

## 2. Results

### 2.1. Structural Water Analysis and Protein Preparation

Structural analysis of the SHP2 catalytic pocket (PDB ID: 4RDD) [20] revealed that its three-dimensional architecture is shaped not only by amino acid residues, but also by a small ensemble of ordered water molecules that mediate key hydrogen bonds (H-bonds) and stabilize local structure. Specifically, four conserved water molecules (W711, W716, W726, and W776) form a highly cooperative hydrogen-bonding network that anchors backbone carbonyls and side chains of Gly427, Ser460, Lys366, and Arg465, thereby defining the geometry and electrostatic landscape of the active site [21]. The conservation of these waters across multiple SHP2 crystal structures underscores their functional relevance and suggests a non-trivial role in ligand recognition. To quantify their contribution, the co-crystallized ligand 3LU was redocked under two conditions: retaining or removing the conserved waters. Inclusion of the water network accurately reproduced the experimental binding pose, yielding a root-mean-square deviation (RMSD) value of 1.43 Å (Figure S1A), well below the 2.0 Å threshold typically accepted for docking validation [22,23,24,25]. In contrast, removal of the conserved waters severely compromised docking reliability, increasing the RMSD to 4.87 Å (Figure S1B), reflecting substantial distortions in binding geometry and disruption of key H-bond interactions. Molecular dynamics (MD) simulations further underscored the structural significance of the conserved water network (Figures S2 and S3). When the conserved waters were included, the ligand remained highly stable, with RMSD values calculated on its heavy atoms after superimposition onto the protein backbone consistently ranging between 2 and 3 Å. In their absence, ligand RMSD increased dramatically to 6–13 Å, reflecting enhanced mobility and destabilization within the binding site (Figure S2). This destabilization was accompanied by elevated protein backbone RMSF values, increased flexibility of the PTP loop, and partial collapse of the catalytic cleft (Figures S3 and S4), collectively highlighting the essential role of conserved waters in maintaining active site integrity and reliable ligand binding. Together, these results establish a fundamental structural principle for SHP2: observed water molecules are integral components of the catalytic pocket, and their explicit inclusion is essential for reliable ligand-binding modeling [26].

### 2.2. Structure-Based Virtual Screening (SBVS)

A chemically diverse screening library comprising 80,867 unique compounds was assembled from the Asinex [27], MolPort [28], and COCONUT [29] databases expanded through ligand preparation to a total of 714,409 discrete ligand states. Structure-based virtual screening [30] was carried out against the SHP2 catalytic pocket with explicit inclusion of the conserved structural water network. The initial screening yielded 63,259 ligand–protein complexes with favorable docking scores. Compounds were initially prioritized relative to the Glide score (G-score) of the reference ligand 3LU (−6.49 kcal/mol), resulting in a focused subset of 133 high-ranking candidates for further evaluation. The distribution of docking scores exhibited a characteristic long-tailed profile, typical of large and chemically diverse libraries. While the majority of compounds displayed moderate predicted affinities, a small fraction achieved highly favorable docking scores. This behavior reflects the properties of a catalytically specialized binding pocket whose geometry and energetics are strongly shaped by conserved structural water molecules and limited steric tolerance [31].

### 2.3. Physicochemical Filtering and Hit Selection

Following docking, the 133 top-scoring ligands were further filtered using physicochemical and structural criteria [32]. To focus on drug-like candidates, compounds exceeding 500 Da, violating Lipinski’s rules [33], or containing PAINS motifs or reactive groups [34,35] were removed, reducing the dataset to 128 candidates. The remaining ligands were carefully inspected using Maestro v. 2020-4 [36] and PyMOL v. 2.6.x [37], focusing on their ability to engage key interactions observed in the reference ligand 3LU complex. Priority was given to poses establishing contacts with key active-site residues, including Gly427, Ser460, Arg465, Thr507, Lys366, Gly464, Ala461, and Gln510, while remaining compatible with the conserved structural water molecules identified during protein preparation. This combined evaluation of physicochemical and structural interactions ultimately highlighted seven ligands with plausible and chemically coherent binding modes (Table 1) [38].

The observed Glide docking scores (−6.76 to −7.61 kcal/mol) are consistent with moderate predicted binding affinities, supporting the prioritization of these compounds for further computational analyses. The small fraction of compounds passing these filters underscores the stringent selectivity of the SHP2 catalytic pocket [39]. Notably, maintaining interactions with both conserved water molecules and key residues appears essential for preserving the structural integrity of the binding site, reinforcing the notion that successful SHP2 binders should accommodate the spatial constraints and the cooperative hydrogen-bonding network characteristic of this catalytically specialized pocket [40].

**Figure 2:**
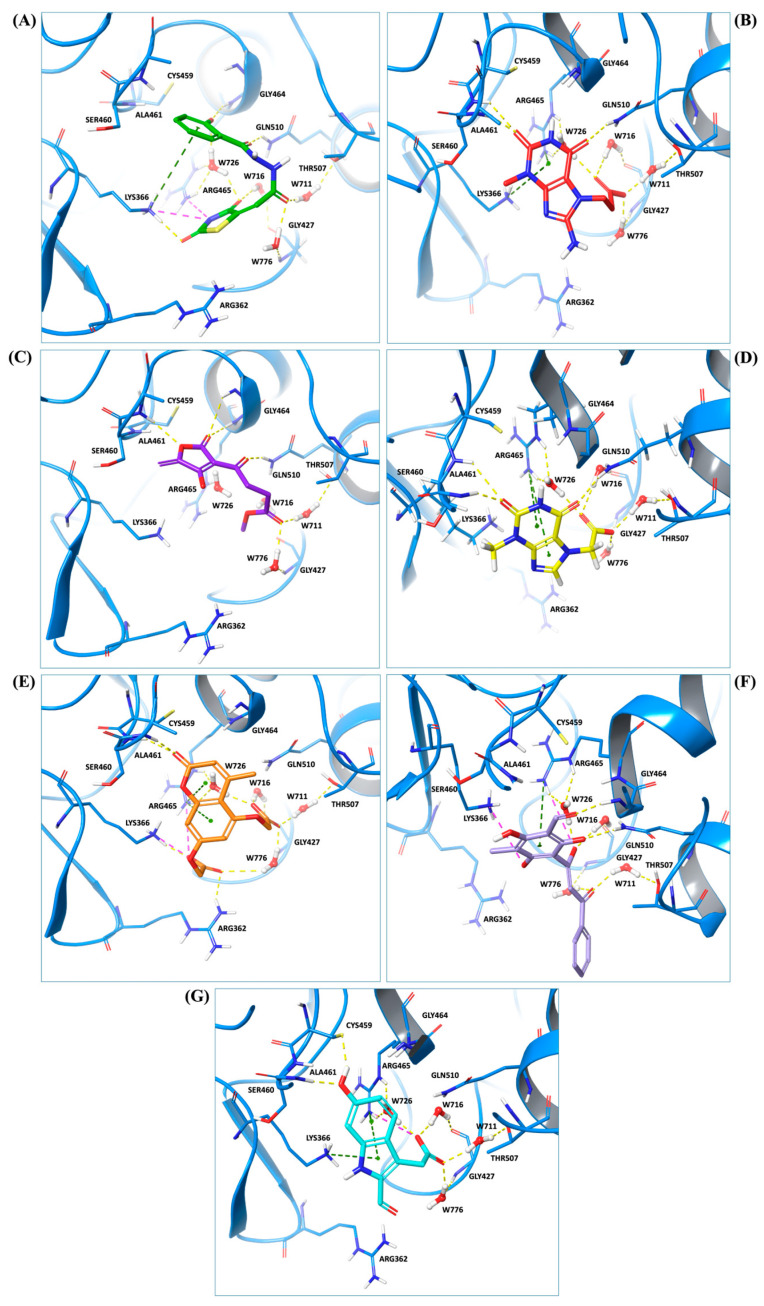
Docking binding modes of the seven selected hits within the SHP2 catalytic pocket: (**A**) compound **1**, (**B**) compound **2**, (**C**) compound **3**, (**D**) compound **4**, (**E**) compound **5**, (**F**) compound **6** and (**G**) compound **7**. The SHP2 protein is shown as cyan ribbons, highlighting the overall fold and the architecture of the catalytic pocket. Ligands and key residues involved in ligand recognition, as well as the conserved structural water molecules, are depicted as sticks.

Predicted binding poses of the seven ligands selected after structure-based virtual screening and physicochemical filtering are shown in Figure 2. All compounds occupy the central region of the SHP2 catalytic pocket, adopting conformations compatible with the conserved architecture of the PTP loop and the surrounding binding cavity. Compound **1** (Figure 2A) engages the SHP2 catalytic pocket through a combination of hydrogen bonding, water-mediated bridges, and hydrophobic/π interactions. Key interactions include carbonyl and hydrazide hydrogen bonds with ARG465, GLN510, THR507, and GLY427, stabilization by structural waters (W711, W716, W726), and hydrophobic or π contacts involving the salicylate aromatic ring and thiazolidine moiety. Compound **2** (Figure 2B) exhibited a distinct scaffold, likely xanthine- or purine-like, while occupying the same SHP2 catalytic pocket as compound **1**. Like compound **1**, it anchors to the protein backbone via structural waters W726 and W716. Notably, compound **2** exhibits a unique binding pattern: its imide-like nitrogens form direct, bifurcated hydrogen bonds with ALA461 and SER460, whereas this ligand exploits the flexibility of its hydrazide linker to interact with GLN510. Compound **3** (Figure 2C) also forms an H-bond with GLN510, as observed for the other ligands, while its acetyl moiety engages in water-mediated bridges with THR507 and GLY427 via W711 and W776, respectively. Additionally, its γ-butyrolactone ring establishes multiple hydrogen bonds with ALA461 and GLY464. Compound **4** (Figure 2D) also displayed a xanthine-like scaffold, similar to that observed for compound **2**. It engages SER460, ALA461, and GLN510 through direct polar contacts, while waters W726, W716, W711, and W776 mediate interactions with ARG465, THR507, and GLY427. Electrostatic complementarity with ARG465 and ARG362, along with hydrophobic contacts from LYS366 and CYS459, further reinforce ligand binding. For compound **5** (Figure 2E), the upper carbonyl forms a H-bond with ALA461, while the central core is anchored by W726 bridging to ARG465. The distal regions are stabilized through the hydration network (W716, W711, W776), mediating interactions with THR507 and GLY427. The fused aromatic system engages in π-stacking or cation–π interactions with ARG465, and the lower polar substituent is stabilized by ARG362. Hydrophobic complementarity from LYS366 and CYS459 further shapes the pocket around the ligand’s scaffold. The central fused-ring system of compound **6** (Figure 2F) engages in cation–π or π-stacking with ARG465, while hydrophobic complementarity is provided by LYS366 and CYS459. Direct H-bonds are formed with SER460 and GLN510, and the ligand’s core and distal tail are stabilized by structural waters W726, W716, W711, and W776, mediating interactions with ARG465, GLY427, and THR507. Finally, compound **7** (Figure 2G) showed a hydroxyl group forming an H-bond with ALA461, while the side-chain of GLN510 anchors the right-side carbonyl or polar substituent. The central bicyclic scaffold engages ARG465 via π-stacking or cation–π interactions, with additional hydrophobic support from LYS366. Structural waters W726, W716, W711, and W776 mediate interactions with ARG465, THR507, and GLY427, stabilizing the ligand’s core and distal regions. Overall, while all seven compounds engage key interactions within the SHP2 catalytic pocket and interact with residues implicated in substrate recognition, the extent, geometry, and integration of direct and water-mediated interactions vary substantially across the series. These differences suggest distinct levels of structural robustness and justified the progression of the seven prioritized hits to the next stages of the workflow. Before energetic and dynamic characterization, the compounds were further examined through an in silico ADME and drug-likeness assessment to verify that the selected candidates also occupied a chemically acceptable developability space.

### 2.4. In Silico ADME Profiling and BOILED-Egg Analysis

**Figure 3:**
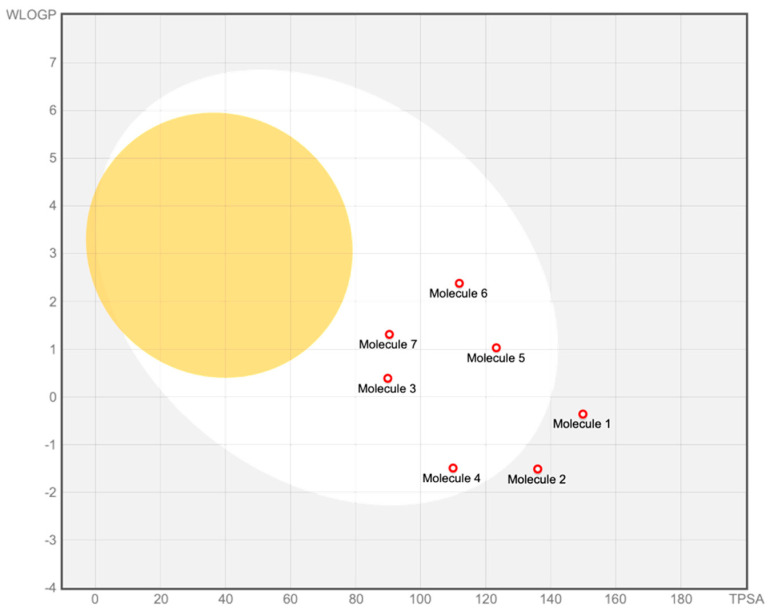
BOILED−Egg diagram generated using SwissADME for the seven SHP2-binding candidates. The plot reports WLOGP versus topological polar surface area (TPSA), highlighting regions associated with passive gastrointestinal absorption (white area) and blood–brain barrier permeation (yellow area). All compounds fall within a chemically feasible ADME space, with none predicted to permeate the BBB.

To complement the structure-based prioritization and to ensure that the selected hits did not exhibit evident developability liabilities, an in silico evaluation of pharmacokinetic and drug-likeness properties was performed for all seven screened compounds using the SwissADME platform. Canonical SMILES were analyzed to estimate key physicochemical descriptors, solubility, lipophilicity, and absorption-related parameters, including the BOILED-Egg model [41], which predicts passive gastrointestinal absorption [42] and blood–brain barrier (BBB) permeation based on molecular polarity and lipophilicity [43]. Overall, the SwissADME analysis [44] indicates that the entire compound set falls within a chemically plausible ADME space, with no significant outliers in terms of molecular weight, polarity, or lipophilicity (Figure 3). Calculated topological polar surface area (TPSA) [45] values span a moderate range (approximately 90–150 Å^2^), while consensus logP values remain within limits compatible with enzyme-targeting small molecules [46]. All compounds display acceptable predicted solubility profiles, and comply with major drug-likeness filters, indicating that the virtual screening did not yield candidates with obvious physicochemical liabilities. According to the BOILED-Egg model, most compounds are predicted to exhibit moderate to high passive GI absorption, whereas none are predicted to permeate the BBB. This behavior is consistent with the relatively polar character of the molecules and supports their suitability for peripheral modulation of SHP2. Importantly, the absence of BBB permeation [47] across the entire set mitigates potential central nervous system exposure, which is unnecessary for the intended therapeutic context. Within this overall ADME landscape, differences among compounds are modest and do not provide an independent basis for ranking or prioritization. Rather, the BOILED-Egg analysis serves as an exclusion and consistency check, confirming that the selected compounds do not differ dramatically in gross physicochemical space and that none shows obvious liabilities that would justify exclusion at this stage of the workflow.

### 2.5. AI-Driven Chemical Space and Novelty Analysis

**Figure 4:**
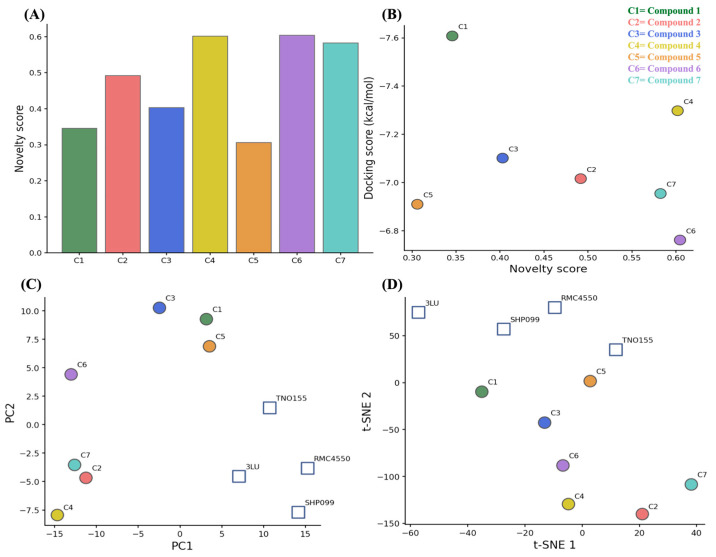
AI-driven chemical space exploration and novelty analysis. (**A**) Novelty scores (QN), defined as one minus the mean cosine similarity between ChemBERTa embeddings of each screened compound and known SHP2 inhibitors, quantify structural divergence from reference chemotypes, with higher values indicating increased novelty. (**B**) Relationship between molecular novelty and predicted binding affinity, shown as a scatter plot of QN values versus docking scores for screened compounds. (**C**) Principal Component Analysis (PCA) and (**D**) t-distributed stochastic neighbor embedding (t-SNE) projections of molecular embeddings. Reference SHP2 inhibitors (squares) cluster within a confined region of chemical space, consistent with their structural similarity, whereas screened compounds (circles) display broader dispersion, highlighting scaffold diversity and occupancy of less represented regions of chemical space.

An AI-driven chemical space analysis, based on transformer-derived molecular embeddings generated with the ChemBERTa model, was performed to evaluate the structural novelty, diversity, and chemical context of the prioritized compounds beyond traditional structure-based metrics [48,49]. This approach provides a contextual and data-driven representation of molecular structures, enabling direct comparison between screened hits and known SHP2 inhibitors (Table S1). Unlike traditional fingerprint-based methods, which rely on predefined structural features, ChemBERTa embeddings capture both local and global molecular patterns learned from large-scale chemical datasets [50,51]. Each molecule was encoded as a 768-dimensional vector derived from the final hidden layer of the pretrained model, enabling a continuous representation of chemical similarity within a high-dimensional latent space. To quantify scaffold originality, a molecular novelty index (QN) was defined as one minus the mean cosine similarity between each screened compound and a set of reference SHP2 inhibitors in the embedding space. As shown in Figure 4A, reference inhibitors displayed QN values close to zero, consistent with their high structural similarity and shared chemotype. In contrast, the screened compounds exhibited a broader distribution of QN values, ranging from moderate to high novelty. Several candidates showed QN values above 0.55, indicating substantial divergence from known SHP2 inhibitor scaffolds.

The organization of the chemical space was further investigated using dimensionality reduction techniques. Principal Component Analysis (PCA) and t-distributed stochastic neighbor embedding (t-SNE) projections (Figure 4C,D) consistently showed a clear separation between reference inhibitors and screened compounds. Reference ligands (3LU, SHP099, RMC4550, and TNO155) formed a compact cluster, reflecting limited scaffold diversity. In contrast, the screened compounds were more broadly distributed, indicating higher structural heterogeneity. Some candidates occupied peripheral or sparsely populated regions of the embedding space, suggesting low similarity to both reference inhibitors and other screened compounds. Consistent clustering patterns observed across PCA and t-SNE projections indicate the stability of the embedding-based representation.

The relationship between structural novelty and predicted binding affinity was also evaluated (Figure 4B). No clear correlation was observed between QN values and docking energies. Highly novel compounds (e.g., compound **4** and compound **7**) retained favorable docking scores, while less novel compounds (e.g., compound **5**) also showed competitive binding energies.

Overall, the results indicate that the screened compounds cover a broader and more diverse chemical space compared to known SHP2 inhibitors, while maintaining comparable predicted binding profiles. These findings support the inclusion of structurally diverse candidates for subsequent evaluation by molecular dynamics simulations.

### 2.6. Outcomes of Molecular Dynamics Simulations

**Figure 5:**
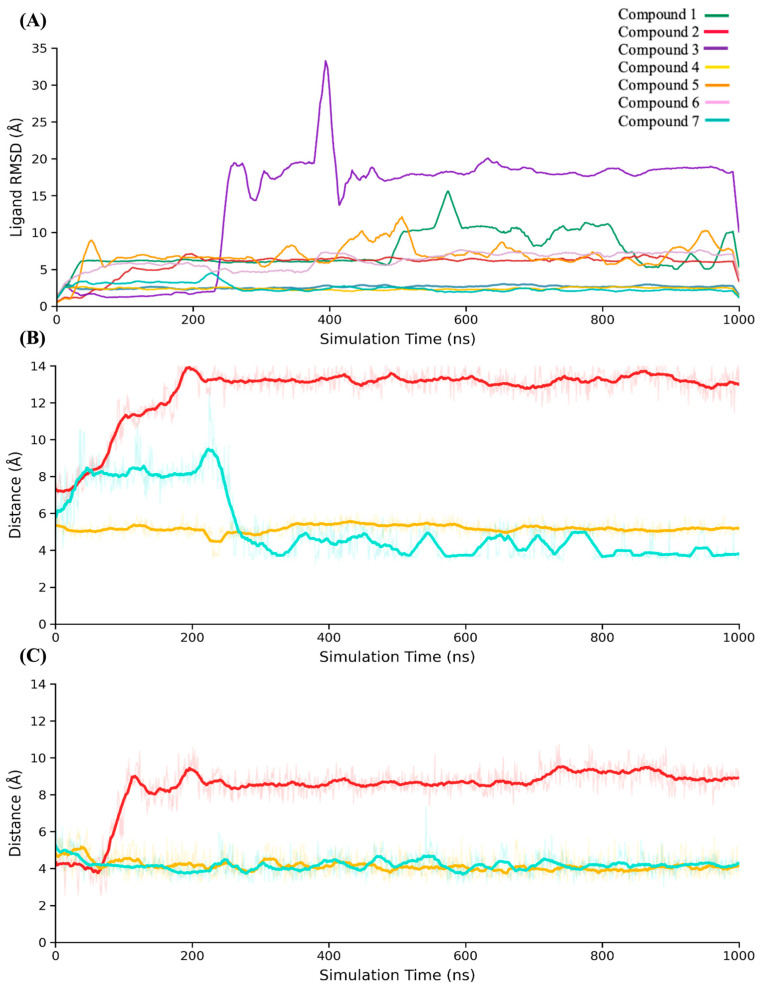
Dynamic analysis of SHP2 hit compounds during 1 μs MD simulations. (**A**) RMSD of the seven hit compounds calculated for ligand heavy atoms after superimposing each protein–ligand complex onto the protein backbone. RMSD provides a measure of ligand binding stability and conformational fluctuations within the catalytic pocket. (**B**) Time-resolved distances between selected ligands (compounds **2**, **4**, and **7**) and the key catalytic residue Ser460. (**C**) Time-resolved distances between the same ligands and the catalytic residue Arg465. These analyses capture both global ligand stability and residue-specific interactions, highlighting differences in binding dynamics among the compounds.

The dynamic behavior of the seven hit compounds identified by SBVS was investigated using the same MD protocol previously employed to evaluate the structural role of the conserved water network within the binding pocket. Throughout the simulations, ligand RMSD with respect to the protein backbone was evaluated to characterize preliminary binding dynamics (Figure 5). Compound **3** exhibited rapid and irreversible RMSD increases (>15–20 Å), indicative of progressive disengagement from the catalytic pocket. Compounds **1** and **5** displayed moderate RMSD fluctuations, remaining associated with the binding site but lacking a well-defined binding pose. Compounds **2** and **6** showed RMSD patterns consistent with confinement within the pocket while exploring alternative orientations and micro-poses. In contrast, compounds **4** and **7** maintained consistently low RMSD values throughout the simulations, retaining binding poses closely aligned with their initial docking configurations. Visual inspection of the trajectories and qualitative assessment of interaction persistence indicate that RMSD alone does not fully capture the mechanistic nuances of ligand binding. Ligands may remain localized within the pocket while undergoing substantial rearrangements of their interactions. To better characterize these dynamics, a residue-level, time-resolved interaction analysis was performed to rationalize the observed behaviors.

To characterize the differences in binding behavior among the hit compounds, we performed a residue-level, time-resolved analysis focused on the distances between each ligand and two key residues in the SHP2 catalytic pocket, Ser460 and Arg465. This approach goes beyond RMSD alone, capturing how ligands engage with critical anchoring points over time, and allowing us to distinguish subtle variations in stability, flexibility, and interaction persistence. By tracking these residue–ligand distances throughout the simulations, we could directly compare the dynamic binding behaviors of structurally similar compounds (such as compound **2** and compound **4**) as well as structurally different compounds with similar dynamic profiles (such as compound **4** and compound **7**). Interestingly, compounds **2** and **4**, despite being structurally similar, exhibit markedly different dynamic behaviors (Figure 5B,C). Compound **4** stabilizes the catalytic pocket, maintaining strong and persistent interactions with key residues Ser460 and Arg465 throughout the simulation, while effectively engaging with the conserved water-mediated network. These stable contacts contribute to a coherent binding mode and minimize fluctuations within the pocket. In contrast, compound **2** shows weaker and less persistent interactions with the same residues, higher local flexibility, and limited involvement in the water network. As a result, although it remains bound, compound **2** adopts a functionally unstable binding mode, with frequent internal rearrangements. Compound **7**, while structurally distinct from compound **4**, displays a dynamic behavior more similar to it than to compound **2**. It retains stable interactions with Ser460 and recurrent contacts with Arg465, although with slightly larger fluctuations compared to compound **4** (Figure 5B,C). This combination of relative stability and flexibility allows compound **7** to perform better than compound **2**, even if it is somewhat less robust than compound **4**. Overall, these observations highlight that structural similarity does not always predict dynamic behavior, and that a ligand’s integration into the conserved interaction network is critical for maintaining a productive binding mode.

### 2.7. MM/GBSA-Based Energetic Evaluation of Prioritized Hits

To assess the molecular dynamics (MD) results using an energy-based criterion, the prioritized compounds were further evaluated through Prime MM/GBSA calculations performed on representative structures extracted from the simulations [52]. This analysis served as a post-MD re-ranking step, providing a comparative estimate of ligand affinity within the SHP2 catalytic pocket while preserving the structural context defined by the conserved water-containing binding site. Based on the RMSD analysis, compound **3** was excluded from subsequent energetic evaluation, as its trajectory revealed progressive displacement from the binding pocket, indicating loss of stable binding during the simulation. Consequently, MM/GBSA calculations were carried out on the remaining six compounds that maintained persistent interactions within the catalytic site.

**Figure 6:**
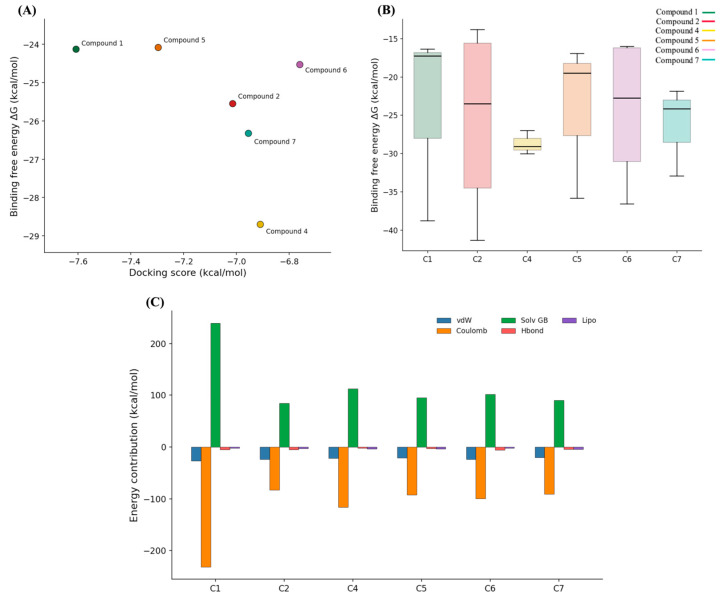
MM/GBSA-based energetic analysis of the six prioritized SHP2 ligands. (**A**) Relationship between docking scores and MM/GBSA binding free energies. Scatter plot comparing the mean docking scores with the mean Prime MM/GBSA energies for the selected compounds. (**B**) Distribution of Prime MM/GBSA binding free energies calculated for the six docking-prioritized compounds, providing an energetic analysis of the initial docking results. (**C**) MM/GBSA energy decomposition for the six prioritized ligands. Average contributions to the binding free energies are shown, including van der Waals, Coulombic, solvation, hydrogen-bonding, and lipophilic terms.

As shown in Figure 6A, the analyzed ligands exhibited heterogeneous MM/GBSA energy distributions, reflecting pronounced variability in predicted binding affinities and highlighting differences in interaction strength and ligand affinity within the target binding pocket. Several molecules, including compounds **2**, **5**, and **6**, exhibited relatively broad ΔG distributions, characterized by large interquartile ranges and extended whiskers. This behavior suggests the presence of multiple conformational states associated with variable energetic quality, reflecting either binding-mode flexibility or a less optimized fit within the catalytic environment. In contrast, compound **4** exhibited one of the most compact energy distributions among the evaluated ligands, centered on comparatively favorable ΔG values. This profile indicates not only predicted stabilization but also enhanced energetic consistency across the sampled MD conformations. Collectively, the MM/GBSA results suggest reproducible energetic compatibility of compound **4** with the SHP2 catalytic pocket. The mean-value comparison presented in Figure 6B further emphasizes the relationship between structural stability and energetic profiles. Although all compounds originated from the same MD-validated set, their average MM/GBSA values did not strictly correlate with the initial docking-based prioritization. Ligands exhibiting comparable Glide scores diverged substantially when evaluated within a dynamic structural framework, confirming that docking scores alone were insufficient to resolve the energetic quality of the complexes. This partial discrepancy underscores the role of MM/GBSA as an intermediate prioritization layer that complements, rather than redundantly reproduces, earlier ranking steps.

Additional insight was gained from the decomposition of MM/GBSA energy terms (Figure 6C). Across the ligand series, binding free energies reflected the expected balance between favorable Coulombic and van der Waals interactions, counteracted by positive solvation penalties. However, the relative contributions of these components varied markedly among compounds. Notably, compound **4** exhibited pronounced electrostatic stabilization, offset by substantial desolvation costs, whereas the remaining ligands displayed more moderate and heterogeneous energetic profiles. Hydrogen-bonding, lipophilic, and van der Waals interactions contributed secondary yet ligand-specific effects, generating distinct energetic fingerprints within the same catalytic site. Overall, the decomposition analysis indicates that individual candidates exploit diverse combinations of polar and nonpolar stabilization, rather than relying on a single dominant binding mechanism.

## 3. Discussion

In this study, we designed a rigorous and fully integrated computational workflow that combines structure-based virtual screening, explicit treatment of conserved structural waters, post-docking energetic re-scoring, AI-driven chemical space analysis, and long-timescale molecular dynamics (MD) simulations to identify and prioritize SHP2-binding small molecules. This multi-step approach addresses key challenges associated with targeting highly dynamic and water-structured phosphatase active sites, a problem recognized in the broader literature on protein tyrosine phosphatases (PTPs) as inherently difficult for traditional ligand discovery strategies due to conformational plasticity and conserved water networks within catalytic domains [53,54,55]. SHP2 has emerged as a central therapeutic target in cancer and developmental disorders, with considerable efforts focused on allosteric and catalytic inhibitors owing to its role in RAS/MAPK signaling. Although several allosteric SHP2 inhibitors have shown promising clinical potential, catalytic-site inhibition remains of considerable interest because it may provide an alternative strategy to overcome resistance mechanisms, target distinct conformational states, and expand the currently limited scaffold diversity of SHP2-directed ligands. At the same time, achieving selectivity within the highly conserved PTP family remains a major challenge. In this context, the explicit consideration of conserved structural waters and residue-specific interaction patterns may contribute to improving target discrimination, although experimental validation against related phosphatases will be required to confirm selectivity. One of the main findings of our work is the critical role of four conserved water molecules (W711, W716, W726, and W776) in maintaining the structural integrity of the SHP2 catalytic cleft [56]. Their inclusion appeared essential both for accurate reproduction of the crystallographic binding mode and for preserving pocket geometry and ligand stability during microsecond-scale MD simulations. This conclusion is consistent with emerging evidence demonstrating that binding site water networks significantly influence protein–ligand recognition and thermodynamic profiling across diverse systems, including ordered and conserved water molecules that stabilize the protein–ligand interface [56]. Recognizing binding site waters as integral components of the active site, rather than interchangeable solvent, aligns with recent advances in computational approaches aimed at explicitly characterizing conserved waters for drug design [57]. As these studies highlight, the inclusion of water networks can meaningfully affect predicted binding energetics and pose stability, a critical consideration when designing ligands for highly hydrated enzyme active sites. Our large-scale virtual screening of over 700,000 compounds, combined with physicochemical and structural filters, yielded a small set of high-confidence hits. Subsequent computational analyses, including AI-driven ChemBERTa analysis, revealed substantial diversity in chemical space occupancy. Although these approaches offered valuable insights into relative binding affinities and structural novelty, explicit-solvent molecular dynamics simulations provided an important evaluative stage, capturing the dynamic behavior of the complexes under near-physiological conditions. Similar integrative strategies that combine docking, MD simulations and free-energy estimates are increasingly reported for SHP2 and other PTPs, reinforcing the utility of such workflows for discriminating among candidate ligands [58]. Among the seven screened candidates, only compounds **4** and **7** maintained a stable and well-defined binding mode throughout the full MD timescale, preserving key interactions with catalytic residues and remaining compatible with the conserved water network. The convergence of structural, energetic, and dynamic evidence for compounds **4** and **7** supports their prioritization as promising SHP2-binding candidates and illustrates the importance of integrating multiple computational layers into hit prioritization workflows. More broadly, the principal contribution of this work lies in the proposed computational framework, which is modular, generalizable, and particularly suited for targets featuring conformational flexibility, complex hydration environments, or limited scaffold diversity. By integrating physics-based simulations with modern AI-driven chemical representations, this workflow enhances both hit prioritization and mechanistic insight, supporting future computational ligand-prioritization efforts in phosphatases and beyond.

## 4. Materials and Methods

### 4.1. Protein Preparation

The crystal structure of the catalytic PTP domain of human SHP2 (*PTPN11*) (PDB ID: 4RDD; residues 262–528) [20] was retrieved from the Protein Data Bank [59] and prepared using the Protein Preparation Wizard implemented in Maestro (Schrödinger Release 2024-2) [60,61]. The selected structure was chosen based on its superior resolution (1.60 Å), providing a highly well-resolved representation of the catalytic pocket. It also includes the co-crystallized active-site ligand 3LU, making it particularly suitable for structure-based modeling and docking protocol validation. Hydrogen atoms were added assuming a physiological pH of 7.0 ± 0.5, missing side chains were rebuilt using Prime, and the structure was subjected to restrained energy minimization with the OPLS4 force field [62] until convergence of the heavy-atom RMSD below 0.3 Å. Four conserved structural water molecules (W711, W716, W726, and W776) were retained based on their consistent presence in the crystallographic structure and their involvement in a hydrogen-bonding network within the catalytic pocket, connecting key residues Gly427, Ser460, Lys366, and Arg465. These water molecules were included in all subsequent docking, molecular dynamics, and free-energy calculations. The co-crystallized ligand 3LU was extracted from the structure, its protonation state verified under physiological conditions, and its crystallographic binding pose was used as a reference for redocking and protocol validation. Since only the catalytic domain was considered in this study, long-range allosteric effects and conformational transitions associated with the regulatory N-SH_2_ and C-SH_2_ domains were not explicitly modeled.

### 4.2. Ligand Dataset Preparation

Three chemical libraries (Asinex, MolPort, and COCONUT) [27,28,29] were collected, merged, and deduplicated to generate a non-redundant compound set. All molecules were prepared using LigPrep v. 4.9 [63,64] to generate energetically minimized three-dimensional conformations. Protonation states were assigned using Epik at physiological pH (7.0 ± 0.5), and relevant stereoisomeric and tautomeric forms were enumerated where applicable. Compounds presenting undefined stereochemistry, reactive functional groups, or chemically unstable or inappropriate moieties were excluded prior to docking. The resulting ligand dataset was used as input for the subsequent structure-based virtual screening.

### 4.3. Molecular Docking

Structure-based virtual screening was performed using Glide v. 8.4 in standard precision (SP) mode [65,66]. Receptor grids were generated by centering the grid box on the co-crystallized ligand 3LU, corresponding to the crystallographic binding site. The grid was defined by setting an inner box of approximately 10 Å × 10 Å × 10 Å, centered on the co-crystallized ligand, ensuring that the ligand centroid remained confined within the catalytic site while allowing sufficient sampling of the surrounding binding cavity. An outer box of 30 Å × 30 Å × 30 Å was additionally applied to encompass the full extent of the binding region and to ensure adequate spatial sampling during docking. The four conserved structural water molecules identified during protein preparation were explicitly retained and included in both grid generation and docking calculations to preserve the native geometry of the catalytic pocket. The co-crystallized ligand 3LU was redocked into the prepared SHP2 structure to validate the docking protocol and to define a system-specific selection threshold. Redocking accurately reproduced the experimental binding mode and yielded a Glide G-score of −6.49 kcal/mol, which was subsequently used as a cut-off for hit selection. Only ligands scoring equal to or better than the reference ligand were retained for further analysis.

### 4.4. Physicochemical Filtering and Structural Inspection

Following molecular docking, candidate ligands were further screened using physicochemical filters aimed at selecting compounds with drug-like properties. Molecules with a molecular weight exceeding 500 Da or violating Lipinski’s rule-of-five criteria were excluded [46,67]. Additional filtering steps were applied to remove structures containing potentially reactive functional groups or substructures associated with assay interference, including known pan-assay interference compounds (PAINS) [34]. Compounds passing these filters were subjected to visual inspection using PyMOL v. 2.6.x [37] to assess the quality and plausibility of their predicted binding poses. Evaluation focused on preservation of key interactions observed for the reference ligand 3LU, including contacts with Gly427, Ser460, Arg465, Thr507, Lys366, Gly464, Ala461, and Gln510, as well as on compatibility with the conserved structural water network within the SHP2 catalytic pocket.

### 4.5. Transformer-Based Molecular Embeddings

Transformer-derived molecular embeddings were generated to provide an orthogonal, data-driven representation of chemical structure independent of classical physicochemical descriptors [68]. Canonical SMILES strings of all ligands were processed using the pretrained ChemBERTa model implemented in the Hugging Face Transformers library (version 4.37) [48]. SMILES sequences were tokenized into learned submolecular units and passed through the ChemBERTa encoder, yielding a 768-dimensional embedding vector extracted from the final hidden layer. No task-specific fine-tuning was performed, as the aim was to characterize global scaffold diversity rather than optimize prediction of a specific molecular property. Dimensionality reduction was performed using principal component analysis (PCA) followed by t-distributed stochastic neighbor embedding (t-SNE) to enable low-dimensional visualization of the chemical space [69,70]. Pairwise cosine similarity values were computed in the embedding space and used to derive quantitative novelty scores for each compound relative to the reference SHP2 inhibitors. All analyses were performed using scikit-learn v. 1.5.x [71].

### 4.6. Quantitative Novelty Score

To quantitatively assess scaffold novelty relative to known SHP2 inhibitors, a molecular novelty metric (QN) was defined based on ChemBERTa embeddings [68]. For each ligand, cosine similarities were computed relative to a curated reference set of experimentally validated SHP2 inhibitors (3LU, SHP099, RMC4550, and TNO155). The novelty score was defined as:

$$QN\;=\;1\;-\left(\frac{1}{n}\right)\sum_{i=1}^{n}\cos\left(\theta_{i}\right)$$

where $\cos\theta_{i}$ denotes the cosine similarity between the embedding of the query ligand and that of the i-th reference compound, and n is the number of reference inhibitors considered. Ligands closely related to known SHP2 scaffolds yield low QN values, whereas compounds occupying more distant regions of the embedding space yield higher scores. This embedding-based metric provides a continuous and conceptually distinct assessment of scaffold novelty compared to traditional fingerprint-based similarity measures, capturing chemical distinctiveness at a representational level learned from large chemical corpora.

### 4.7. Molecular Dynamics Simulations

To assess the stability of docking-derived poses under explicit-solvent conditions, microsecond-scale molecular dynamics (MD) simulations in explicit solvent were performed. Protocol validation was first carried out using four reference systems: apo SHP2 with conserved structural waters, apo SHP2 without conserved structural waters, 3LU-bound SHP2 with conserved structural waters, and 3LU-bound SHP2 without conserved structural waters. The validated protocol was subsequently applied to the screened protein–ligand complexes while retaining the conserved structural water molecules to characterize binding stability and interaction persistence. All simulations were carried out using Desmond v. 6.3 [72]. Protein–ligand complexes were solvated in an orthorhombic simulation box using the TIP5P water model [73], selected for its accurate representation of hydrogen-bonding patterns, particularly in the presence of conserved structural waters. The OPLS4 force field was applied to both protein and ligands [62]. Systems were neutralized by addition of Na^+^ and Cl^−^ counterions and equilibrated using the default Desmond relaxation protocol. Production simulations were conducted in the NPT ensemble at 300 K and 1 atm, employing the Nosé–Hoover thermostat and the Martyna–Tobias–Klein barostat. Long-range electrostatic interactions were treated using the particle mesh Ewald (PME) method. Protocol validation was performed using apo and ligand-bound SHP2 systems with and without conserved structural waters. These reference simulations provided a benchmark for evaluating pocket stability, ligand retention, and water-mediated interaction networks. Following protocol validation, each of the seven prioritized hit compounds was simulated in complex with SHP2 for 1 μs under identical conditions. These simulations were used to evaluate ligand stability, binding mode persistence, and interaction patterns within the catalytic pocket. Trajectory analyses were carried out using the Simulation Interaction Diagram (SID) toolkit in Maestro, complemented by in-house Python scripts based on MDAnalysis and MDTraj.

### 4.8. MM/GBSA Free-Energy Calculations

Binding free-energy calculations were performed using the Prime MM/GBSA module [52]. For each protein–ligand complex, 100–200 snapshots were extracted from the equilibrated portion of the MD trajectories to represent the dominant conformational states sampled during production simulations. Molecular mechanics energies were computed for the complex, isolated receptor, and isolated ligand using the VSGB 2.0 implicit solvent model. Binding free energies were calculated according to:

$${\Delta G}_{\mathrm{bind}}=E_{complex}-\left(E_{receptor}+E_{ligand}\right)$$

Average ${\Delta G}_{\mathrm{bind}}\;$ values were obtained by ensemble averaging over all selected frames.

## 5. Conclusions

Our integrated computational workflow combining virtual screening, explicit water modeling, AI-driven chemical space analysis, and long-timescale molecular dynamics enabled the identification and prioritization of SHP2-binding candidates. Conserved water molecules appeared to play a critical role in maintaining catalytic pocket integrity and ligand stability, with compounds **4** and **7** emerging as the most promising candidates. This modular approach provides a generalizable framework for targeting flexible, water-structured enzymes and may support the rational design and prioritization of structurally novel inhibitors.

## Figures and Tables

**Table 1:** IUPAC Name, 2D structure and G-Score of the 7 selected hits.

Compound	IUPAC Name	2D Structure	G-Score (kcal/mol)
Compound **1**	N′-{2-[(5E)-2,4-dioxo-1,3-thiazolidin-5-ylidene]acetyl}-2-hydroxybenzohydrazide	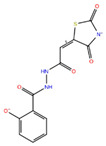	−7.61
Compound **2**	3-(8-amino-3-methyl-2,6-dioxopurin-7-yl)propanoic acid	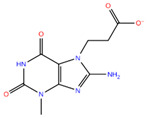	−7.02
Compound **3**	methyl 4-(4-hydroxy-5-methylidene-2-oxo-2,5-dihydrofuran-3-yl)-4-oxobutanoate	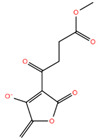	−7.10
Compound **4**	2-(3-methyl-2,6-dioxo-2,3,6,7-tetrahydro-1H-purin-7-yl)acetate	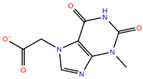	−7.30
Compound **5**	2-[5-(carboxymethoxy)-4-methyl-2-oxochromen-7-yl]oxyacetic acid	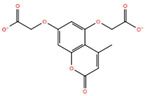	−6.91
Compound **6**	2,4,6-trihydroxy-3-methyl-5-(3-oxo-3-phenylpropanoyl)benzaldehyde	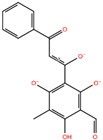	−6.76
Compound **7**	2-(2-formyl-6-hydroxy-1H-indol-3-yl)acetic acid	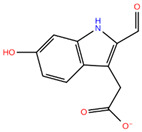	−6.95

## Data Availability

The original contributions presented in this study are included in the article/Supplementary Material. Further inquiries can be directed to the corresponding author.

## Supplementary Materials

The following supporting information can be downloaded at: https://www.mdpi.com/article/10.3390/ph19050706/s1, Figure S1. Impact of conserved structural water molecules on the accuracy of ligand redocking in the SHP2 catalytic site; Figure S2. Ligand RMSD over 1μs MD simulations of the crystallographic complex with and without conserved water molecules. Ligand RMSD values were calculated on the ligand heavy atoms after superimposition onto the protein backbone; Figure S3. Backbone RMSF profiles of SHP2 in the presence and absence of conserved structural water molecules; Figure S4. Effect of conserved water molecules on PTP loop dynamics; Table S1. Reference ligands used for similarity and AI-based analyses.

## Abbreviations

The following abbreviations are used in this manuscript:

PTPs	Protein tyrosine phosphatases
SBVS	Structure-based virtual screening
MD	Molecular dynamics
H-bonds	Hydrogen-bond
RMSD	Root-mean-square deviation
G-score	Glide Score
BBB	blood–brain barrier
TPSA	topological polar surface area
QN	molecular novelty index
PCA	Principal Component Analysis
t-SNE	t-distributed stochastic neighbor embedding
