# Supplementary material for: A Multi-Step Computational Workflow for Screening and Prioritizing SHP2-Binding Molecules
Source: Pharmaceuticals (Basel). 2026 Apr 30;19(5):706. doi: 10.3390/ph19050706 (PMC13209867; doi:10.3390/ph19050706)
Supplement: Supplementary file 1 [file pharmaceuticals-19-00706-s001.zip › pharmaceuticals-4285842-supplementary.pdf]

Supplementary Information

A Multi-Step Computational Workflow for Screening and Prioritizing SHP2-Binding Molecules

Marina Bilotta[1], Roberta Rocca[12], Stefano Alcaro[12]

[1] Dipartimento di Scienze della Salute, Università "Magna Græcia" di Catanzaro, Italy

[2] CRISEA Centro di Ricerca e Servizi Avanzati per l'Innovazione Rurale, Italy

Supplementary Figures

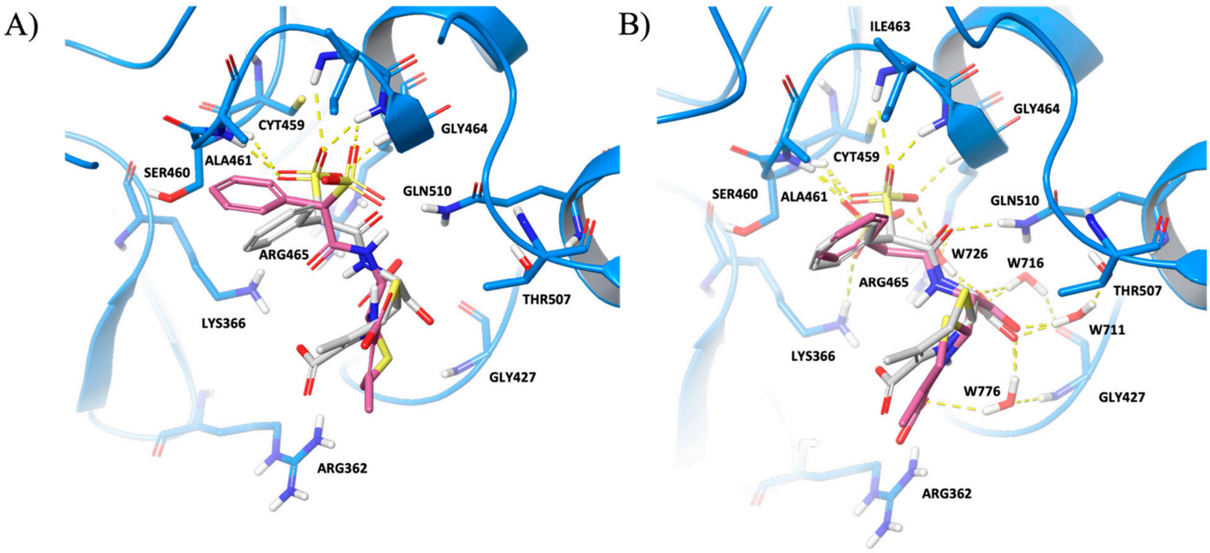

Figure S1. Impact of conserved structural water molecules on the accuracy of ligand redocking in the SHP2 catalytic site. The co-crystallized ligand (grey) is superimposed with the corresponding redocked poses (pink) obtained in the absence **(A)** and presence **(B)** of conserved structural water molecules. The protein is represented as a blue cartoon, key residues are shown as sticks, and hydrogen bonds and polar interactions are depicted as yellow dashed lines.

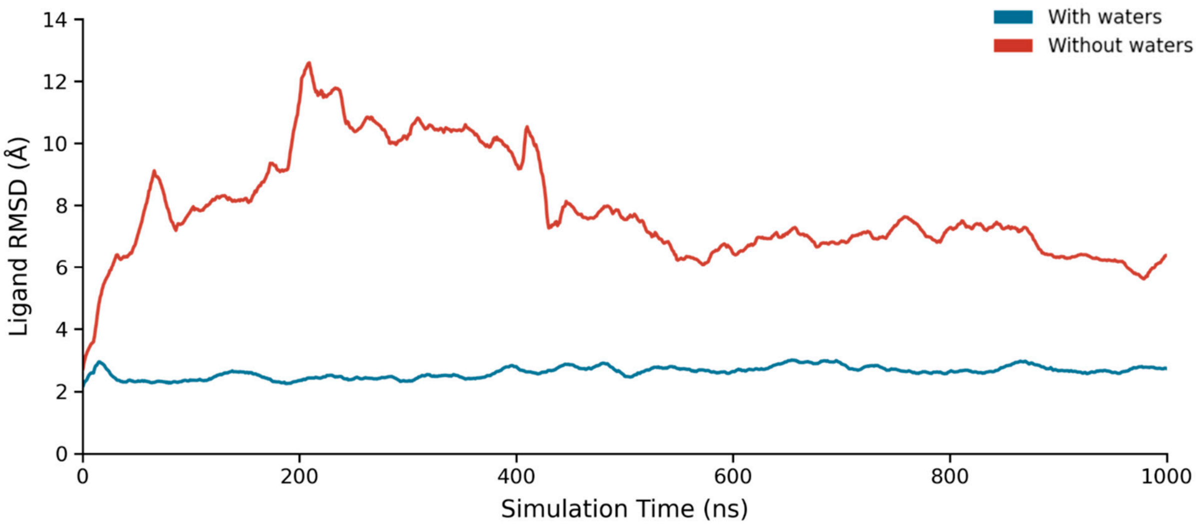

Figure S2. Ligand RMSD over 1μs MD simulations of the crystallographic complex with and without conserved water molecules. Ligand RMSD values were calculated on the ligand heavy atoms after superimposition onto the protein backbone.

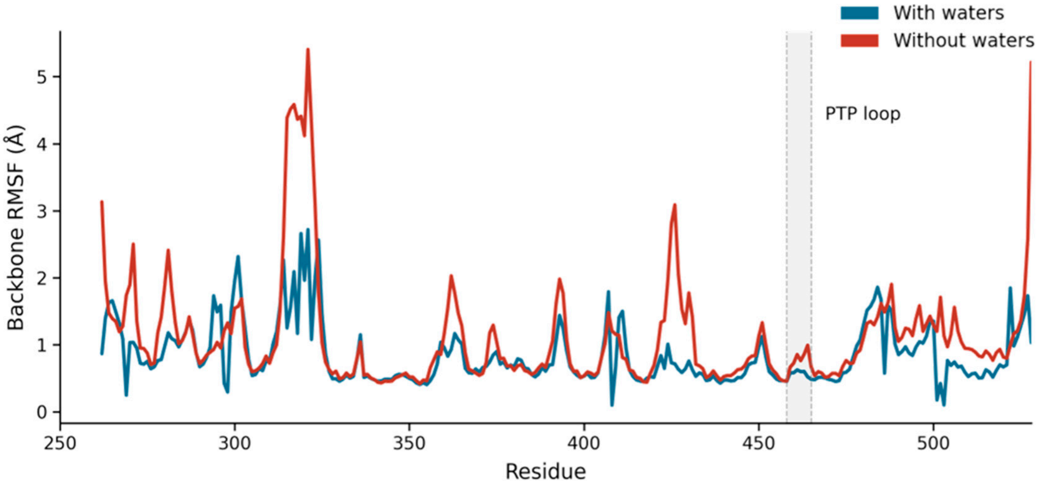

Figure S3. Backbone RMSF profiles of SHP2 in the presence and absence of conserved structural water molecules. Root-mean-square fluctuation (RMSF) of SHP2 backbone atoms (residues 262–528), calculated from molecular dynamics simulations, is shown for simulations performed with conserved water molecules (blue) and without them (red).

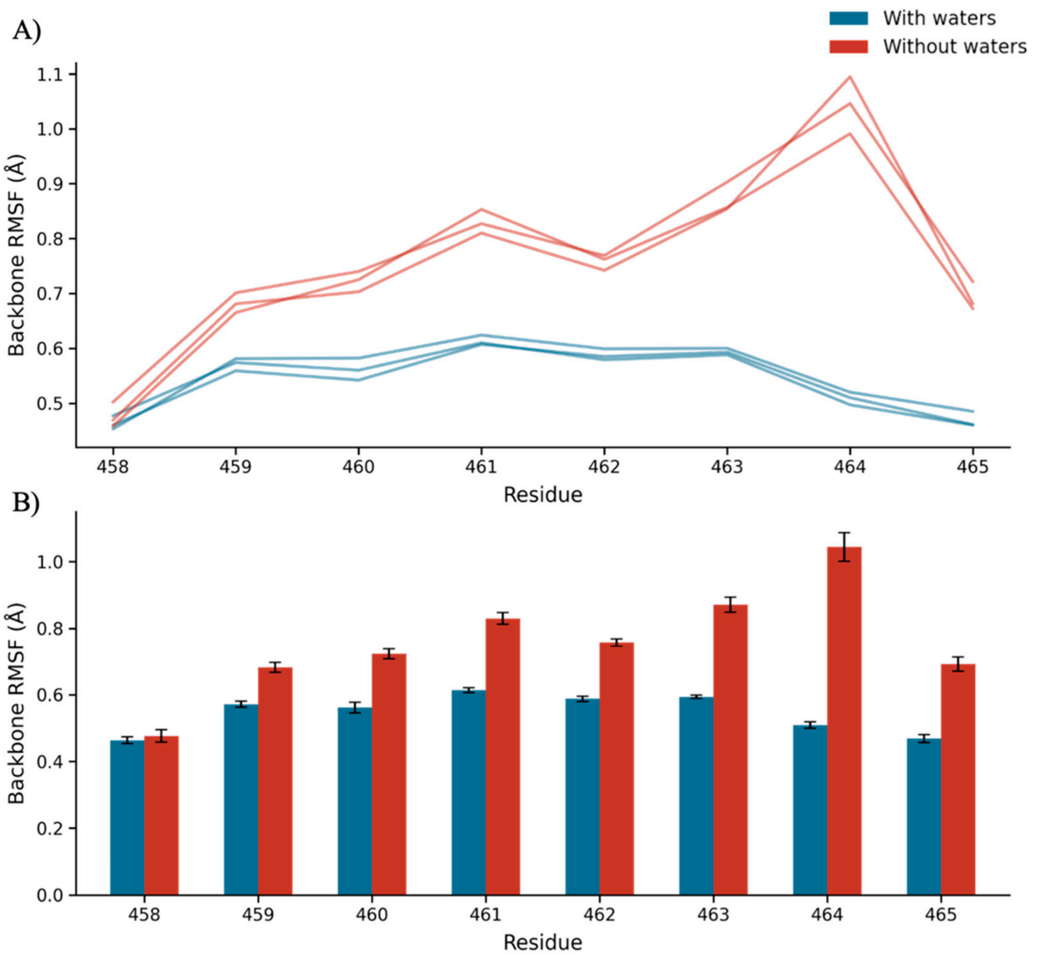

Figure S4. Effect of conserved water molecules on PTP loop dynamics. Backbone RMSF of the PTP loop region (residues 458–465) calculated from three independent molecular dynamics simulations in the presence (blue) and absence (red) of conserved structural water molecules. **A)** Overlay of RMSF profiles from the three replicas, showing high reproducibility within each condition and clear separation between datasets, indicating increased loop flexibility upon water removal. **B)** Average RMSF values ± standard deviation across replicas, highlighting a consistent increase in backbone fluctuations for all residues when conserved waters are absent, with the largest effect at residue 464.

Reference ligand	2D structure
**3LU**	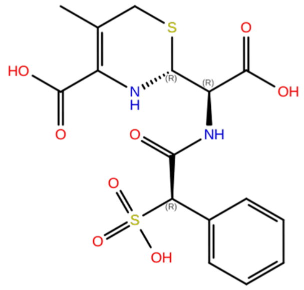
**SHP099**	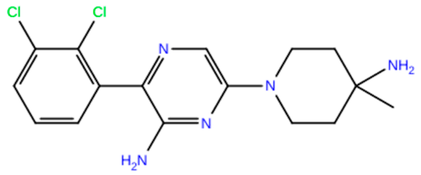
**RMC4550**	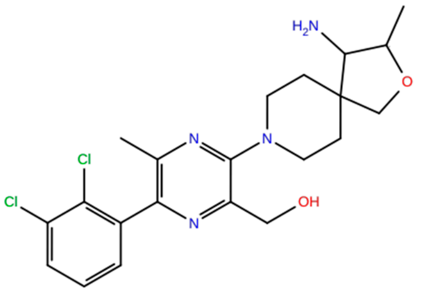
**TNO155**	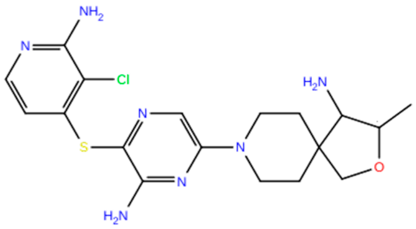

Table S1. Reference ligands used for similarity and AI-based analyses. The table reports the reference ligands considered in this study, including the co-crystallized ligand 3LU and the

known SHP2 inhibitors SHP099, RMC4550, and TNO155, together with their corresponding 2D chemical structures. These compounds were used as benchmarks for chemical space comparison, similarity assessment, and AI-based analyses to contextualize the predicted ligands within the landscape of known SHP2 binders.
